# Assessment of oral findings of dental patients who screen high and no risk for obstructive sleep apnea (OSA) reporting to a dental college - A cross sectional study

**DOI:** 10.5935/1984-0063.20180021

**Published:** 2018

**Authors:** Sonal Sadashiv Kale, Pradnya Kakodkar, Sahana Hegde Shetiya

**Affiliations:** 1 Dr. D.Y. Patil Vidyapeeth, Dr. D.Y Patil Dental College and Hospital, Department of Public Health Dentistry - Pune - Maharashtra - India.

**Keywords:** Obstructive Sleep Apnea, Risk Factors, Diagnosis, Oral, Surveys and Questionnaires, Dentists

## Abstract

To assess the oral findings of patients who screen high and no risk for obstructive sleep apnea (OSA) reporting to outpatient department of a dental college. **Methods:** Patients coming to dental Out Patient Department (OPD) were screened using STOP questionnaire and were categorized into high (n=130) and no risk (n=130) OSA groups. BANG (body mass index, age, neck circumference and gender) was recorded for both the OSA risk group patients. Following this oral and general examination was performed using predetermined criteria for temporomandibular disorder (TMD), Angle’s Class of Malocclusion, maxillary arch constriction, facial profile, Mallampati score for uvula, tongue size, depth of palatal vault and periodontitis. Chi-squared statistics was applied to know the significant difference among the two groups. Multivariate logistic regression model was run by including the significant variables. **Results:** 94 females and 166 males were present in the study with a mean age of 43.67±11.89 in both the risk groups. All the variables except Angle’s class of malocclusion and periodontitis showed significant difference among high and no risk OSA groups. Logistic regression confirmed that neck circumference, Class 3 or 4 Mallampati score, large tongue and deep palatal vault were commonly observed among high risk OSA group and were independent risk factors for developing high risk of OSA. **Conclusion:** Neck circumference>40cm, large tongue, Class 3 or 4 Mallampati score and deep palatal vault were found to be independent predictors of developing high risk of OSA. Dentist can play a vital role in screening such patients as he comes in close vicinity of oral cavity and thus can refer the patients to sleep physician to promote interdisciplinary approach.

## INTRODUCTION

Sleep plays a vital role in good health and well being throughout the life. Many people experience trouble in sleeping which may be because of stress or other factors and is usually temporary, but becomes a concern when it occurs repeatedly thus indicating a sleep disorder. Sleep disorders like bruxism and obstructive sleep apnea (OSA) are of great concern to the dentist^1^. Dental sleep medicine is an emerging branch which deals with these sleep disorders by providing treatment with oral appliances^2^.

Among all sleep disorders, OSA has the highest mortality rate if not diagnosed and treated^3^. It is characterized as complete cessation of breathing for 10 seconds or more during sleep due to complete or partial pharyngeal obstruction leading to frequent arousal during sleep and excessive day time sleepiness^4^^,^^5^. Usually, when the pharyngeal muscles relax and collapse back during sleep it does not lead to upper airway obstruction but, in OSA patients this collapse causes obstruction of upper airway leading to difficulty in breathing and sometimes skipping in the breathing cycle^6^.

During this skip of breath (absence of breathing seconds) the oxygen supply to all the organs is arrested leading to organ cell damage and thus OSA has been linked to systemic diseases like hypertension, stroke, myocardial infarction, congestive heart failure, intolerance diabetes, depression and excessive daytime sleepiness^7^^,^^8^. All these systemic diseases have some or the other oral manifestations like periodontitis, dental caries, and other oro-facial problems^9^^-^^11^. As patient coming to dental clinic will have oral problems which may be linked with OSA thus, presence of any of this history in dental patients should precipitate questions regarding sleep disorders.

The gold standard for diagnosing OSA is polysomnography which records Apnea-Hypoapnea Index (AHA-I) whose value ≥5 per hour confirms about OSA^12^. Though polysomnography is the gold standard it is not always feasible as the person has to sleep entire night in the sleep clinic and moreover patients are not aware about the consequences of sleep disorders which restricts them from getting this expensive test done^13^.

Many questionnaires^14^ have been developed and validated to screen for OSA risk patients like STOP, Berlin, Epworth sleepiness scale, STOP-BANG questionnaire and Pittsburg sleep quality index^15^ (in children). Among these, STOP questionnaire is the most commonly used questionnaire having sensitivity of 72%^16^, which increases to 83.6% after including body mass index (BMI), Age, Neck circumference and Gender (STOP-BANG questionnaire)^16^.

As dentists examines the oral cavity and also have a clear view of oropharynx they can play a vital role in screening the patients with sleep disorders using validated questionnaires and further referring the patient to specialist department for final diagnosis, thus promoting the interdisciplinary approach. Against this background the present study was undertaken to screen the patients reporting to dental outpatient department (OPD) of Dr. D.Y. Patil Dental College and Hospital for OSA through questionnaire followed by performing an oral examination of the screened high and no risk OSA patients.

## METHODS

A cross-sectional study was conducted at Dr. D.Y. Patil Dental College and Hospital, Pimpri, Pune from May-August 2017 for assessing the oral findings of OSA risk patients reporting to Out Patient Department (OPD) of this college. Ethical approval was obtained from Institutional Ethics Committee prior to starting the study.

Patients above 18 years, providing written informed consent, willing to answer STOP questionnaire for initial screening and undergoing further examination were included in the study while those undergoing orthodontic treatment, with history of orthognathic surgeries, having adverse habit of drinking alcohol, edentulous patients, patients coming with an oral acute infection on the day of examination and undergoing treatment of OSA or snoring were excluded.

Initial pilot study gave 9.52% prevalence for high risk OSA patients based on STOP^16^ questionnaire criteria. Patients responding ‘YES’ for 2 or more questions(STOP) out of 4 were considered to be at high risk and those responding ‘NO’ to all the questions were considered at no risk for OSA. Thus based on this prevalence, and keeping the ratio of 1:1, sample size of 130 each was calculated for high and no risk OSA patients.

Patients in the study were recruited using convenience sampling. The study initiated by first screening the patients with STOP questionnaire followed by grouping them into high and no risk patients and then performing further general and oral examinations of both the groups. General examination included screening the patient with BANG questionnaire^16^ for recording BMI, age, neck circumference and gender.

Following this oral examination was performed based on predetermined criteria for temporomandibular disorders (TMD present or absent) adopted by Sanders et al.^6^, facial profile (concave straight or convex) by imagining a line passing through glabella, subnasale and the pogonion^17^, Uvula for Mallampati score (Class 1,2,3 and 4)^18^, tongue for lateral indentations (large or normal tongue size)^19^, maxillary arch constriction (present or absent) by using Chadda’s index^20^, for dental attrition (present or absent) occlusal and incisal surfaces of teeth were examined for loss of enamel or dentin, molar relation as per Angle’s Class of Malocclusion (Class 1,2 or 3), depth of palatal vault (shallow, moderate or deep palate)^21^, and periodontitis (present or absent) was examined based on WHO 1997 criteria^22^ for CPI index (codes 3 and 4) along with Loss of attachment (codes 1, 2, 3 and 4). All these parameters were chosen based on the literature review regarding oral findings of OSA patients^1^^,^^6^^,^^19^^,^^21^^,^^23^.

**Figure 1:**
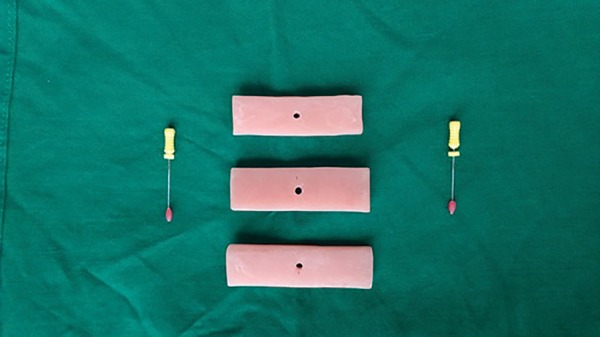
Acrylic palatal bars for measuring the depth of palatal vault.

For recording the palatal depth a novel technique was introduced wherein a palatal bars (Figure 1) with sizes of 45mm, 47mm and 55mm in width and 5 mm in height with the thickness of 2mm were fabricated using hot cure acrylic. This bar was placed over the 1^st^ maxillary molars and a perpendicular distance from the palatal bar till the palatal vault was recorded by inserting a reamer with stopper through the hole at the centre of the bar. Wax was applied over the tip of the reamer so as to prevent any damage to the palatal tissue by the reamer while recording.

### Statistical analysis

Chi-square test was applied between the groups for each variable to know if there was any significant difference between the groups. Following this a multivariate logistic regression was performed on SPSS 21 by including those variables in the model which showed statistical significant difference by chi-square test. The level of significance was fixed at *p*<0.05.

## RESULTS

260 patients (130 high risk and 130 no risk patients) aged 21-72 (43.67±11.89) years participated in the study with 94 females and 166 males. As it was an age-matched study so the mean age in both high and no risk OSA group was same. The parameters measured among the two groups are presented in Table 1. It was noted that BMI, large neck circumference, presence of attrition, presence of TMD, convex facial profile, class 3 and 4 Mallampati score for uvula, large tongue, narrow maxillary arch and deep palatal vault showed statistically significant difference.

**Table 1:** Oral and general findings of patients among high and no risk OSA groups.

Variables	No risk OSA	High risk OSA	X^2^ value	*p* value
BMI <35kg/m^2^	124	114	4.966	0.026*
BMI >35kg/m^2^	6	16		
Neck circumference <40cm	115	77	28.75	0.000*
Neck circumference >40cm	15	53		
Dental attrition absent	72	55	4.449	0.035*
Dental attrition present	58	75		
TMD absent	106	90	5.306	0.021*
TMD present	24	40		
Angle's Class 1 or Class 3 malocclusion	125	117	3.82	0.051
Angle's Class 2 malocclusion	5	13		
Narrow maxillary arch absent	106	85	8.700	0.003*
Narrow maxillary arch present	24	45		
Straight or Concave facial profile	125	115	5.41	0.02*
Convex facial profile	5	15		
Class 1 or Class 2 Mallampati score	110	47	63.81	0.000*
Class 3 or Class 4 Mallampati score	20	83		
Large tongue absent	108	44	64.87	0.000*
Large tongue present	22	86		
Shallow or moderate palatal vault	97	57	25.48	0.000*
Deep palatal vault	33	73		
Periodontitis absent	96	83	3.031	0.82
Periodontitis present	34	47		

BMI=body mass index; OSA=obstructive sleep apnea; TMD= temporomandibular disorder

* significance at *p*<0.05, X2 =chi-squared test value.

Thus variables showing significant difference were included in logistic regression model and Odds Ratio (B exp) was obtained for each variable by adjusting for all other variables (Table nº.2). According to the multivariate logistic regression large neck circumference, Class 3 and 4 Mallampati score for uvula, large tongue and deep palate were perfectly associated with OSA which denotes that the risk of being into high risk of OSA is 2.9 times more in participants with neck circumference >40cm as compared to the participants with neck circumference <40cm, 2.78 time more in participants with Mallampati score of Class 3 or Class 4 as compared to the participants with Mallampati score of Class 1 or Class 2, three times more in participants having large tongue as compared to participants not having large tongue and 2.2 times more in participants having deep palatal vault as compared to participants having shallow or moderate palatal vault.

**Table 2:** Unadjusted and adjusted odds ratio of variables included in logistic regression model.

Variables	Unadjusted OR	Sig (*p*<0.05)	Adjusted OR	Sig (*p*<0.05)	95% C.I. for	EXP(B)
					Lower	Upper
BMI	4.966	.026	.382	.160	.100	1.461
Neck Circumference	28.756	.000	2.905	.017*	1.207	6.990
Facial Profile	5.417	.020	.668	.537	.186	2.400
Mallampati score (Uvula)	63.814	.000	2.785	.047*	1.015	7.639
Large Tongue	64.873	.000	3.032	.038*	1.063	8.649
Narrow Maxillary Arch	8.700	.003	1.456	.356	.656	3.232
Deep Palate	25.484	.000	2.215	.032*	1.070	3.232
Constant			.247	.000		

BMI=body mass index;

* significance at *p*<0.05, adjusted odds ratio for one variable obtained by adjusting all other variables.

## DISCUSSION

As the dentist routinely examines oral cavity there are few findings which may be linked with OSA. The present age and gender matched study was conducted to assess and compare these oral findings of high risk OSA patients with that of no risk OSA patients. In the present study, 83 males and 47 females were identified as high risk for OSA. The results were similar with other studies^24^^,^^25^ indicating males at higher risk for OSA as compared to females due to the difference in fat deposition area over the body.

The unadjusted odds ratio revealed that both BMI >35kg/m^2 ^and neck circumference >40cm were the risk factor for OSA but after adjusting for all other variables the difference was not significant for BMI while it was significant for neck circumference. This may be because BMI gives an overall impression of person’s obesity and not specific information related to the localized fat deposition around the neck which may act as a precise predictor for OSA.

Comparable results were reported by the studies done by Nuckton et al.^25^ wherein odds ratio for BMI dropped from 1.1 to 0.3 after adjusting for all other variables. While Sharma et al.^26^ and Ruangsri et al.^21^ reported contrasting results. Whereas, the literature^21^^,^^27^^,^^28^ supports the findings that higher neck circumference is observed significantly more in OSA patients compared to non OSA patients along with one review^24^ concluding that neck circumference is a good predictor for OSA over BMI because of localized fat deposition. The increase in fat deposition in neck region leads to enlargement of upper airway structures further leading to narrowing and collapsing of airway space and difficulty in breathing.

Literature^6^^,^^29^^,^^30^ reveals that attrition and temporomandibular disorders are the consequences of OSA rather than risk factors and thus they were not included in logistic regression model. But odds ratio showed that participants with attrition and TMD were significantly more in high risk OSA group compared to no risk OSA group. A study^29^ stated alike findings and explained that when tongue collapses posteriorly causing reduction in airway space body may activate its inbuilt protective mechanism wherein it moves the mandible forward unconsciously to make space for air in upper airway region leading to attrition of teeth and this action caused by forward movement of mandible repeatedly leads to excessive strain over TMJ causing TMD in the long run.

In the present study, participants with Angle’s Class 2 malocclusion were not significantly more among OSA high risk group as compared to OSA no risk group. The results were similar with the studies done by Al-Madani et al.^27^ and Triplett et al.^31^ which did not find Angles Class 2 malocclusion to be a risk factor for OSA. Contrast results were mentioned by Banabilh et al.^32^.

A series of review published in 2012 stated few of the craniofacial risk factors for OSA, and narrow maxillary arch was one amongst the factors^24^. In the present study the presence of narrow maxillary arch was significantly more among high risk OSA group as compared to no risk OSA group. Alike results have been reported in the literature^27^^,^^33^. The inter-premolar and inter-molar distance is significantly less among OSA patients compared to non-OSA patients thus reporting a causal relationship between them^33^. While on the other side Banabilh et al.^34^ states a contrasting result.

Angle’s Class II malocclusion, facial profile and narrow maxillary arch are related with each other. Class II malocclusion is more likely to cause convex facial profile and a narrow maxillary arch. The narrow maxillary arch may reduce the upper airway space leading to increased risk of difficulty in breathing due to collapsed tongue along with reduced tongue space which further contributes to risk of developing OSA.

The Mallampati score gives a picture of the amount of tissue present in the posterior oropharyngeal region. Class 3 and 4 score suggests crowding in the pharyngeal region making it difficult to breath while sleeping when the tongue collapse posteriorly. The present study reported a significant difference among number of participants with Class 3,4 and Class 1,2 among the two OSA risk groups.

Similar results have been reported in other studies^18^^,^^21^^,^^25^^,^^35^^,^^36^ indicating it as independent risk factor for developing OSA. Even after adjusting for all other variables in the present logistic regression model the difference was significant. This was opposing with the results of one another study^28^ after adjusting for BMI and neck circumference who suggested that controlling BMI and neck circumference (fat deposition) may control OSA risk.

The muscle activity decreases during sleep and so does the tongue activity causing it to collapse and mask the posterior area including tonsillar pillars and uvula^19^. In the present study number of participants with large tongue were more in OSA risk group compared to non OSA risk group. Concordance results were found with Ruangsri et al.^21^ and Weiss et al.^19^. The odds ratio was 3.03 in the present study after adjusting for all other variables which was contradicting another study^28^ in the literature.

Participants with deep palate in the present study were significantly more among the high risk compared to no risk OSA group which was contrasting with a study^21^ in which the number of participants with high palatal height were almost same giving a non-significant difference between the OSA and non-OSA group. Periodontitis variable as a symptom of OSA did not show a significant difference amongst the OSA risk groups which was in accordance with Loke et al.^37^ study while it was contrasting with other studies^23^^,^^38^. This may be because periodontitis is a multi-factorial disease and OSA alone cannot explain the presence or absence of it.

In the present study, after adjusting for all other variables in logistic regression model neck circumference >40cm, Mallampati score of Class 3 or Class 4, large tongue and deep palatal vault were significant risk factors. Among the four significant factors the highest odds ratio was obtained for large tongue followed by neck circumference, Mallampati score and deep palate. Our results are in confirmation with the results in the literature^25^^,^^26^^,^^28^^,^^36^^,^^39^.

If the patients are not aware about the sleep disorders, consequences of this on their life and are seen taking snoring lightly which is one of the sign of sleep disorders, it is necessary that dentist takes up this charge of screening his day-to-day patients when he notices any of the oral findings related to OSA and educate the patients accordingly to take treatment of the same.

The present study developed a novel technique of measuring palatal vault depth by fabricating an acrylic bar. This technique spared the necessity of making an impression of each patient to obtain its cast for measuring palatal vault depth. Thus direct measurement could be taken on the patients reducing the time of the dentist and expenses of materials.

The study has few limitations: Firstly, instead of gold standard (PSG) a questionnaire was used to categorize patients into high and no risk OSA groups. For initial screening the STOP questionnaire and further to assess the BMI and neck circumference as the dependent variables for Obstructive sleep apnea risk, the BANG checklist was considered. PSG is not possible everytime for community type studies with large sample size. This is a lacunae in our study. Altenatives like the use of home sleep test (HST) or portable sleep monitors can be considered for the future studies. Secondly, since the study was conducted in a hospital setup there is a chance of inducing hospital based bias.

This study can throw light on the important role a dentist can play to identify the signaling oral findings of high risk OSA and refer those patients to sleep physicians. In the long run, early detection of OSA patients by the dentists will result in good prognosis.

## CONCLUSION

To conclude, neck circumference >40cm, large tongue, Mallampati score of Class 3,4 and deep palatal vault were found to be independent predictors of developing high risk for OSA. As a dentist examines the patient’s oral cavity and comes across any of these finding he should enquire about patient’s sleep history and screen them with available validated questionnaire and make necessary referral if required. He should thus contribute towards educating his patients about the sleep disorders and their consequence.

For this, dentists should keep their knowledge up to date regarding these diseases and conditions through dental education programs because even the lack of up to date knowledge may lead to many cases being undetected and treated which may increase the vulnerability of patient’s life as OSA is linked with systemic diseases and is considered to be fatal.
